# Comment on “Nontuberculous Mycobacteria Isolation from Clinical and Environmental Samples in Iran: Twenty Years of Surveillance”

**DOI:** 10.1155/2015/327068

**Published:** 2015-09-08

**Authors:** Eduardo Hernández-Garduño

**Affiliations:** Unidad de Enseñanza e Investigación Centro Oncológico Estatal (COE), Instituto de Seguridad Social del Estado de México y Municipios (ISSEMyM), 50180 Toluca, MEX, Mexico

A review of 20 studies and 14 case reports published in a recent issue of the journal from 14 provinces in Iran on clinical and environmental isolates of nontuberculous mycobacteria (NTM) showed* M. fortuitum* (229/997; 23%) as the most prevalent species from 1992 to 2014 [1]. The majority of NTM were collected from respiratory samples. The primary method of NTM detection was by culture using the Löwenstein-Jensen media. Molecular methods were also used depending on the particular laboratory. From 1992 to 2006, most laboratories used traditional methods for NTM detection whether from clinical or environmental samples and from 2009 to 2014, advances in laboratory technology using a combination of traditional and molecular methods resulted in the identification of more species of NTM.

With the small sample size limitation, trend analysis showed a significant increase in NTM detection rates during the study period. The authors concluded that the increase in NTM in Iran is attributable to the implementation of enhanced molecular techniques that have improved the detection coupled with the enhanced awareness of NTM in the clinical setting. Apparently clinical and radiographic information of individuals was not available; therefore, it was not possible to establish whether the increase in NTM detection was associated with an increase in the number of patients with NTM disease or in the number of people “colonized” with NTM.

The American Thoracic Society criteria of NTM disease include microbiological, radiographic, and clinical criteria [2]. In a previous population-based study from British Columbia, Canada, we also found an increase of NTM isolates over time mostly in people “colonized” with* Mycobacterium avium* complex (MAC) which coincided with changes in laboratory technology [3]. Even though clinical and radiographic information was not available in our study, there was a decrease over time on the number of patients treated for NTM disease. All positive AFB and culture results for mycobacteria must be reported to the TB Control Department of the British Columbia Centre for Disease Control in Vancouver (BCCDC) in the province of British Columbia, Canada. Also, all TB medications dispensed in the province are recorded at BCCDC; therefore all patients receiving treatment for NTM disease in the province were included in our study. Though clinical and radiographic review was not available for our study, the group of “treated” patients was used as a surrogate of NTM disease as all treated patients are assessed and treated by respirologists. To confirm whether the incidence of NTM disease is increasing in specific setting, future epidemiological studies should include the laboratory technique used for identifying each NTM species along with clinical and radiographic assessment of patients to establish whether they have the disease or are just colonized.
